# Dietary patterns in clinical subtypes of multiple sclerosis: an exploratory study

**DOI:** 10.1186/1475-2891-8-36

**Published:** 2009-08-10

**Authors:** Geeta SM Ramsaransing, Sanne A Mellema, Jacques De Keyser

**Affiliations:** 1 Department of Neurology, University Medical Center Groningen, University of Groningen, Groningen, The Netherlands; 2 Department of Neurology, Universitair Ziekenhuis Brussel, Vrije Universiteit Brussel, Brussels, Belgium

## Abstract

**Backround:**

Multiple sclerosis is a neurodegenerative disorder with a wide range in disease course severity. Many factors seem to be implicated in multiple sclerosis disease course, and diet has been suggested to play a role. Because limited data is present in the literature it was investigated whether variations in dietary intake may be related to the severity of the disease course in multiple sclerosis.

**Methods:**

Using a food diary during 14 days, the dietary intake of 23 nutrients and vitamins was measured in patients with primary progressive (n = 21), secondary progressive (n = 32), and benign multiple sclerosis (n = 27) and compared to each other. The intake measured was also compared to the intake of the Dutch population and to the recommended daily allowance.

**Results:**

Compared to the other MS groups, the secondary progressive MS patients had a lower intake of magnesium, calcium and iron. The total group of MS patients had, compared to the Dutch population, a lower intake of folate, magnesium and copper and a lower energy intake. Compared to the daily recommended allowance, the MS patients had a lower than recommended intake of folic acid, magnesium, zinc and selenium.

**Conclusion:**

Magnesium, calcium and iron intake may possibly be related to MS disease progression, and should receive further attention. This is important because no effective neuroprotective treatment for MS patients is available.

## Background

Multiple sclerosis (MS) is a common neurodegenerative autoimmune mediated disorder of the central nervous system. The typical clinical presentation of MS is that of a relapsing-remitting disorder. The majority of MS patients accumulates irreversible physical disability in time and switch to a secondary progressive form in which there is a continuous downhill course that may still be accompanied by overlapping relapses. However, a subgroup of patients with relapsing-remitting MS shows a benign course with no disease progression and minimal disability decades after the first manifestations. Eventually, these patient may switch to a progressive state.

A systematic review of longitudinal studies revealed that about 25% of patients with MS have a benign disease course [1]. There is also a primary progressive form of MS in which patients show progression of disability from the onset of disease with or without overlapping relapses.

There is as yet no clear explanation for the heterogeneity in disease course. Genetic factors may contribute to the clinical phenotype, as well as environmental factors. An important environmental factor is diet. Experimental, epidemiological and clinical studies suggest that nutritional factors may influence the incidence as well as the course of MS [2].

For MS patients disease modifying drugs, such as beta interferons, are used in an attempt to modify the disease course. These medications are prone for side effects, and should be used cautiously. Patients may also be treated unnecessary while there is a prospect of a benign course. If dietary intake can be related to disease course severity, it might be a way to influence the course of MS as an alternative or complementary strategy. However, the relation between dietary intake and MS disease course is, up to this date, not clear [2].

Therefore it was decided to investigate whether there are differences in nutritional intake between patients with different MS disease courses that may play a role in disease course severity. Also the nutritional intake was compared to the Dutch population and to the recommended daily intake to find possible differences or deficiencies that need attention.

## Methods

### Patients

The study was approved by the medical ethics committee of the University Medical Centre Groningen. All patients provided written informed consent prior to inclusion in the study. A total of 80 patients with MS were enrolled: 27 with a benign course, 32 with secondary progressive MS, and 21 with primary progressive MS. Benign course in MS was defined as an Expanded Disability Status Scale (EDSS) score of 3.0 or less, despite at least 10 years of disease duration, and without disease progression [1].

### Dietary assessment

The patients were extensively informed on how to keep a food diary at home. A food diary was developed in close collaboration with the dietary counselor of the University Medical Centre Groningen. Each patient recorded on a daily basis all items of food and drinks consumed in a log-book that was presented to them. For each day, the log-book had the following sections: at breakfast, during the morning, at lunch, during the afternoon, at dinner, and during the evening. Per section a list of commonly consumed items was presented that could be marked/numbered. For example: the section breakfast had a list containing '...slices of white bread, ...slices of whole wheat bread,..biscuits,...crackers', etc. Then the kind of butter was asked for, then the topping of the bread (what kind and amount of meat, what kind and amount of cheese, and so on).

For accuracy, the dietary assessment took place over a period of 14 consecutive days, and outside holidays or other festivities such as Easter or Christmas. Patients were asked to provide all possible information about preparation, i.e, one egg for breakfast, boiled or baked. Also the use of food supplements was scored. The presence of co-morbidity was scored as well (i.e. hypertension). The information in the log-books were then entered in a database. Computerized analyses were subsequently performed using a dietary software program based on the validated and widely used Dutch nutrient database [3] which covers detailed information on energy and nutrients calculated per 100 grams of 1603 food items. Based on the diary, total daily intake amounts of 23 nutrients were calculated for all food items consumed. Also, the body mass index (BMI) was assessed by dividing the body weight by the square of the body height.

### Serum levels

Vitamin A (measured as retinol), vitamin C (measured as ascorbine-acid), vitamin B_1 _(measured as thiamindiphosphate), vitamin B_2 _(measured as flavin-adeninedinucleotide in EDTA-anti-coagulated whole blood), vitamin B_6 _(measured as pyridoxal-5'-phosphate in EDTA-anti-coagulated whole blood) were analyzed by HPLC. Serum vitamin B_12 _and serum folate were analyzed with an immunofluorometric method (Autodelfia™, Wallac Oy, Turku, Finland). Vitamin E was measured as the sum of α-tocoferol and γ-tocoferol. Magnesium was determined with the Xylidyl blue method, calcium with the CPC method and iron with the ferene method (all Ecoline^® ^MEGA, Holzheim, Germany).

### Comparison of food intake to Dutch population/recommended daily allowance

Dietary values of daily nutritional intake in the Dutch population were retrieved from the Dutch Organization for Applied Scientific Research [4]. The values were obtained from an at random sample of 5.958 individuals in the Dutch population with an age below 75 years, and were presented in age subgroups. For an accurate assessment we compared the daily nutritional intake of the patients from our study group with the corresponding subgroups.

### Statistical Analysis

Differences between the groups were assessed by ANOVA and Student's t-test (two-tailed); nonparametric tests were used when variables did not have normal distribution. Only when results between the three groups were significant, further comparisons were done using Dunn's multiple comparisons test. Because of the number of items measured, the Bonferroni correction was applied to correct for significant results by coincidence. The results are presented as means ± SD, or median [range]. Pearson and Spearman correlation analysis test was performed to study correlation. All statistical tests were interpreted at the 5% two-tailed significance level.

## Results

The results are presented in table 1 and Additional file 1. All patients filled in the food diary with accuracy. There were no patients with special diets such as vegan, vegetarian or gluten free. There were no differences between the BMI between the groups (p > 0.05), nor were there differences in the number of patients with over- or underweight (p > 0.05, data not shown). The most striking result was an approximately 20% lower intake of magnesium (p = 0.009) and a 15% lower intake of calcium (p = 0.03) in patients with secondary progressive MS compared to patients with either benign or primary progressive MS. Per group only a few patients used food supplements that were mostly herbal based. No relation could be found between the use of supplements and disease course, nor between the dietary intake and the presence of co-morbidity (data not shown). Serum levels were within normal range, did not differ between the groups, and did not correlate with intake (data not shown, all p > 0.05). Compared to Dutch population the intake of the following nutrients was significantly lower in MS patients: protein (p < 0.002), SAFA (p = 0.002), MUFA (p < 0.002), total fat (p = 0.005), cholesterol (p = 0.01), folic acid (p < 0.002), magnesium (p = 0.002) and copper (p = 0.01). Compared to the daily recommended allowance it was found that the intake of folic acid, magnesium, zinc, copper and selenium in MS patients is below the daily recommendation (Additional file 1.). Total energy intake was lower in the MS group than in the Dutch population (p < 0.05).

**Table 1:** Patient characteristics

**Multiple sclerosis**	Benign*	**Secondary progressive**	**Primary progressive**
Number	27	32	21
Age (years, mean ± SD)	49.7 ± 10.3	50.7 ± 9.9	52.4 ± 8.7
Gender (F/M)	18/9	21/11	14/7
Disease duration (years, mean ± SD)	20.7 ± 8.2	20.2 ± 8.6	12.0 ± 4.8
EDSS score (median, range)	2.0 (0.0 – 3.0)	7.0 (4.5 – 8.5)	6.0 (2.5 – 8.0)
BMI (± SD)	23.8 ± 5.2	25.6 ± 5.8	24.9 ± 5.6

F: female; M: male; SD: standard deviation; EDSS: Expanded Disability Status Scale; BMI: Body Mass Index (kg/l^2^).

*Defined as an Expanded Disability Status Scale (EDSS) score of 3.0 or less, despite at least 10 years of disease duration. Score 0 = no disability, 10 = death [1].

## Discussion

Patients with secondary progressive MS have a more severe disease course than patients with a benign course. The stepwise accumulating disability following exacerbations is believed to be caused by axonal injury in the focal white matter lesions in the central nervous system through inflammatory mechanisms [5,6]. A slowly progressive axonal degeneration may be the mechanism responsible for the progressive worsening in progressive multiple sclerosis (both secondary and primary).

### Magnesium

Magnesium is neuroprotective in models of white matter ischemia and traumatic axonal injury [7], and improves neurological outcome in patients with neural damage due to lacunar infarctions [8]. The exact mechanism of neuroprotection by magnesium remains unclear, but may be relevant for explaining a possible inverse association between magnesium intake and tissue damage in MS. This neuroprotective property of magnesium in lesions affecting the white matter of the central nervous system may be relevant for explaining a possible inverse association between magnesium intake and tissue damage in MS. Magnesium regulates more than 300 metabolic processes [9] yet the exact mechanisms providing axonal protection remain uncertain. Those that may be implicated in playing a protective role include energy metabolism, membrane stability, protein and DNA synthesis [10], and inhibition of the inducible enzyme nitric oxide synthase (iNOS) [11]. The appearance of iNOS in astrocytes and macrophages in lesions of MS gives rise to the production of high amounts of nitric oxide (NO) as well as superoxide radicals. These radicals can promote oligodendrocyte injury, demyelination, and axonal damage [12]. Axons may degenerate because NO can inhibit mitochondrial respiration, leading to intraaxonal accumulation of Na^+ ^and Ca^2+ ^ions [13]. Inhibition of iNOS by magnesium would therefore be considered neuroprotective.

Interestingly, a small post mortem study showed that magnesium concentrations in central nervous tissue and visceral organs obtained from MS patients were significantly lower than seen in controls [14]. The most marked reduction of Mg content was observed in CNS white matter including the demyelinated plaques of MS patients. Whether or not these significantly lower Mg contents found in CNS and visceral organs of MS patients may play an essential role in the demyelinating process remain unclear, but this lower Mg content might have a relation with a reduced nutritional intake as found by our results. We found a lower intake of magnesium in the total MS group compared to the Dutch population (p = 0.001).

### Calcium

On a high calcium diet, vitamin D supplementation prevents experimental autoimmune encephalomyelitis [15], which serves as an animal model for the inflammatory component of MS. However, this protective effect was abolished with a low calcium diet, and restored with a high calcium diet [16], indicating that the positive effect of vitamin D can be increased by calcium. Interestingly, a pilot study in a small group of MS patients found that dietary supplement with magnesium, calcium and vitamin D resulted in a significant decrease in relapse rate [17]. A low calcium intake may therefore be considered negative to MS disease course, and may be reflected in our results, since the primary progressive MS patients have a lower calcium intake than the patients with a benign disease course.

### Iron

Iron is considered an important factor in the pathogenesis of multiple sclerosis [18]. Iron may cause neuronal damage by acting as a catalyst for hydroxyl radical formation [19], leading to lipid peroxidation [20] and stimulating oxidative damage to proteins [21]. Limitation of iron intake may provide protection from developing experimental autoimmune encephalomyelitis [22]. The difference in iron intake between the secondary and primary progressive patients found in this clinical study suggests that iron may play a role in MS disease progression.

### Folic acid

Compared to the Dutch population, protein, cholesterol and folic acid intake was significantly lower in the MS group. Folates and vitamin B_12 _have fundamental roles in the central nervous system at all ages, especially the methionine-synthase mediated conversion of homocysteine to methionine, which is essential for nucleotide synthesis. Deficiencies in folic acid (as vitamin B_6 _or vitamin B_12_) are associated with elevated plasma levels of homocysteine. Elevated plasma homocysteine levels in patients with secondary progressive MS compared to healthy controls have been found [23], suggesting a possible link between the vitamin B status and MS progression. In an earlier study we found higher homocysteine levels in MS patients as compared to healthy controls, but this was unrelated to disease progression, or deficiencies in vitamins B_6_, B_12 _or folic acid [24].

### Other nutrients

As others [25], we could not find an association between antioxidant intake (vitamin C, E, and selenium) or serum levels and MS disease progression.

In lipid research, limitation of saturated fatty acid intake [26], and supplementation with unsaturated fatty acids in combination with more vegetables [27] would favour prognosis in relapsing-remitting MS. This may be related to anti-inflammatory properties of the omega-3-fatty acids [28]. We did not find a difference in the fatty acids intake between the MS groups and no relation to disease course severity.

### Serum levels

We found no difference in the serum levels of the vitamins or trace elements between the MS groups. This may raise the question if the found differences in intake are biologically relevant. Serum levels of several vitamins and elements are maintained in a homeostasis to a certain degree, even if there is a shortage in the body or an excess. For example; magnesium is mobilised from bone if necessary to maintain a steady serum level. This implicates that, although serum levels are within normal range, the metabolism at tissue level may be altered and lead to biological relevant effects.

In general, supplementation of nutrients may especially be needed in the presence of disease, where an altered metabolic state may enhance the need for certain nutrients, vitamins or trace elements. Compared to the daily recommended allowance, the intake of folic acid, magnesium, zinc, copper and selenium were lower in our MS patients (Additional file 1.) and total energy intake (kcal) as well. Also total energy intake of the MS patients was lower than the energy intake of the Dutch population. This may reflect the higher risk for malnutrition in MS patients, because of handicap, obesity or medications [2], and may suggests a need for dietary counseling.

There are limitations to our study that can serve as a base for future studies.

The results point towards magnesium, calcium and iron intake as possible candidates to be implicated in MS disease course. Ideally, a prospective dietary study in patients with recently diagnosed MS should be undertaken to find out if the differences in nutritional intake found can be confirmed to be involved in disease course modulation.

In MS patients, disability may influence dietary intake, since the more disabled may be more dependent on other persons to cook for them. Also, after receiving the diagnosis MS, patients may alter their dietary habits in an attempt to influence the disease. It is difficult for controlling these factors, but in this study the patients stressed that they did not change their diet over the last years nor were they on special diets. Furthermore, the BMI did not differ between the groups, suggesting that there seems no tendency for over- or underweight related to disease course.

Although it is difficult to interpret the findings of any nutritional survey because of the complex interaction with nutrients and human metabolism, and potential biases that can be difficult corrected for, the findings of this study may suggest a role for food intake in MS. Because the intake of several nutrients on the MS patient group was below the daily recommended allowance, we feel that MS patients should be made aware of possible nutritional deficiencies. To obtain an optimal nutritional intake, our MS patients may be advised to take dietary supplementation of magnesium, zinc, copper and selenium. A source of magnesium is bread, grain products, vegetables and diary products. Zinc can be found in meat, bread, cheese, nuts and shell-fish. Copper is present in vegetables, bread, meat, fruit and cacao products. Selenium can be found in meat, bread and fish. For the patients with a (secondary) progressive course of MS, additional calcium supplements may be advised (milk, cheese, vegetables, nuts). These findings are of importance because up to this date, no effective neuroprotective therapy is available for MS patients.

## Conclusion

From this exploratory study we found differences in magnesium, calcium and iron intake between subgroups of MS patients that may possibly be related to MS disease progression. This is important because no effective neuroprotective treatment is currently available for MS patients.

## Abbreviations

MS: Multiple Sclerosis.

## Competing interests

The authors declare that they have no competing interests.

## Authors' contributions

GSMR: study design, data gathering, stastistical analysis, preparation manuscript. SAM: study design, data gathering. JDK: general supervision.

## Supplementary Material

Additional file 1 **Total daily nutritional intake (mean ± SD) in the MS study groups and Dutch population, including Recommended Daily Allowance**. This table displays the daily intake of various nutrients, trace elements and kcal in 3 subgroups of MS patients, the total MS group and the Dutch population. Click here for file
