# Supplementary material for: Dietary patterns in clinical subtypes of multiple sclerosis: an exploratory study
Source: Nutr J. 2009 Aug 10;8:36. doi: 10.1186/1475-2891-8-36 (PMC2731789; doi:10.1186/1475-2891-8-36)
Supplement: Additional file 1 — Total daily nutritional intake (mean ± SD) in the MS study groups and Dutch population, including Recommended Daily Allowance. This table displays the daily intake of various nutrients, trace elements and kcal in 3 subgroups of MS patients, the total MS group and the Dutch population. [file 1475-2891-8-36-S1.doc]

**Additional file 1-** Total daily nutritional intake (mean ± SD) in the MS study groups and Dutch population, including Recommended Daily Allowance

| **Nutrient** | **BMS**  **(n=27)** | **SPMS**  **(n=32)** | **PPMS**  **(n=21)** | **MS subgroup comparison**  **p-value^*)^** | **MS (n=80)** | **Dutch population** | **MS patients versus Dutch population**  **p-value *)** | **Recommended**  **Daily**  **Allowance** |
| --- | --- | --- | --- | --- | --- | --- | --- | --- |
| Protein (g) | 73 ± 22 (292 ± 88 kcal) | 65 ± 12 (260 ± 48 kcal) | 70 ± 12 (280 ± 48 kcal) | ns | 69 ± 16 (276 ± 64 kcal) | 80 ± 13 (320 ± 52 kcal) | < 0.001 | 11% ** (275 kcal) |
| SAFA (g) | 30 ± 10 (270 ± 90 kcal) | 27 ± 8 (243 ± 72 kcal) | 28 ± 8 (252 ± 72 kcal) | ns | 28 ± 9 (252 ± 81 kcal) | 35 ± 13 (315 ± 169 kcal) | 0.001 | < 10% **) (< 250 kcal) |
| MUFA (g) | 25 ± 9 (225 ± 81) | 21 ± 6 (189 ± 54 kcal) | 23 ± 8 (207 ± 72 kcal) | ns | 22 ± 8 (198 ± 72 kcal) | 31 ± 12 (279 ± 108 kcal) | < 0.001 | > 10% **) (> 250 kcal) |
| PUFA (g) | 15 ± 6 (135 ± 36) | 13 ± 4 (117 ± 36 kcal) | 14 ± 1 (126 ± 9 kcal) | ns | 14 ± 5 (126 ± 45 kcal) | 16 ± 8 (144 ± 72 kcal) | ns | 5.3 - 10% **) (132 – 250 kcal) |
| Total fat (g) | 78 ± 27 (702 ± 234 kcal) | 67 ± 19 (603 ± 171 kcal) | 72 ± 3 (648 ± 27 kcal) | ns | 72 ± 23 (648 ± 207 kcal) | 89 ± 32 (801 ± 288 kcal) | 0.005 | 30 - 35% **) (750 – 875 kcal) |
| Linoleic acid (g) | 12 ± 5 (108 ± 45 kcal) | 11 ± 4 (99 ± 36 kcal) | 11 ± 1 (99 ± 9 kcal) | ns | 11 ± 5 (99 ± 45 kcal) | 14 ± 7 (126 ± 63 kcal) | 0.05 | > 2% **) (> 50 kcal) |
| Cholesterol (mg) | 165 ± 59 (1 ± 1) kcal) | 169 ± 109 (1 ± 1 kcal) | 184 ± 79 (2 ± 1 kcal) | ns | 172 ± 86 (2 ± 1 kcal) | 218 ± 79 (2 ± 1 kcal) | 0.01 | < 300 mg (< 3 kcal) |
| Carbohydrate (g) | 221 ± 111 (884 ± 444 kcal) | 174 ± 46 (696 ± 184 kcal) | 184 ± 79 (736 ± 316 kcal) | ns | 210 ± 77 (840 ± 308 kcal) | 232 ± 72 (928 ± 288 kcal) | ns | 55% **) (1375 kcal) |
| Total kcal | 1978 ± 202 | 1659 ± 110 | 1765 ± 100 | ns | 1865 ± 156 | 2177 ± 157 | < 0.05 | 2500 |
| Magnesium (mg) | 307 ± 81 | 254 ± 49 | 319 ± 76 | 0.009 ^a)^ | 286 ± 71 | 337 ± 93 | 0.001 | 300 mg |
| Calcium (mg) | 967 ± 255 | 826 ± 282 | 992 ± 244 | 0.03 ^a)^ | 917 ± 271 | 1010 ± 416 | ns | 800 mg |
| Iron (mg) | 10.1 ± 4 | 9.2 ± 3 | 11.1 ± 3 | 0.04 ^b^) | 10 ± 3 | 11.4 ± 3 | ns | 14 mg |
| Zinc (mg) | 8.4 ± 2.3 | 7.6 ± 1.6 | 8.4 ± 1.7 | ns | 8.09 ± 1.88 | 8.24 ± 3.4 | ns | 15 mg |
| Copper (mg) | 1.50 ± 0.79 | 1.29 ± 0.43 | 1.36 ± 0.64 | ns | 1.39 ± 0.6 | 1.11 ± 0.3 | 0.01 | 1.5 - 3.5 mg |
| Selenium (μg) | 38 ± 15 | 35 ± 13 | 38 ± 16 | ns | 37 ± 14 | 45 ± 21 | ns | 50 - 150 μg |
| Folic acid (μg) | 133 ± 49 | 131 ± 31 | 150 ± 52 | ns | 137 ± 44 | 260 ± 91 | < 0.001 | 150 - 300 μg |
| Vitamin A (μg) | 768 ± 336 | 788 ± 260 | 939 ± 369 | ns | 821 ± 322 | 936 ± 912 | ns | 800 - 1000 μg |
| Vitamin B_1_ (mg) | 1.07 ± 0.43 | 1.14 ± 0.72 | 1.14 ± 0.43 | ns | 1.12 ± 0.57 | 1.31 ± 1.10 | ns | 0.8 - 1.2 mg |
| Vitamin B_2_ (mg) | 1.42 ± 0.36 | 1.29 ± 0.43 | 1.50 ± 0.36 | ns | 1.39 ± 0.40 | 1.55 ± 0.58 | ns | 1.2 - 1.6 mg |
| Vitamin B_6_ (mg) | 1.50 ± 0.43 | 1.29 ± 0.29 | 1.50 ± 0.43 | ns | 1.41 ± 0.39 | 1.63 ± 0.59 | ns | 1.2 - 1.6 mg |
| Vitamin B_12_ (μg) | 3.5 ± 1.5 | 3.6 ± 3.6 | 3.6 ± 1.9 | ns | 3.57 ± 2.58 | nd | nd | 1.5 - 2.5 μg |
| Vitamin C (mg) | 63 ± 32 | 83 ± 47 | 106 ± 121 | ns | 82 ± 72 | 86 ± 53 | ns | 55 - 70 mg |
| Vitamin D (μg) | 3.5 ± 1.4 | 3.1 ± 1.4 | 3.4 ± 1.1 | ns | 3.3 ± 1.33 | 3.7 ± 2.2 | ns | 0 - 5 μg |
| Vitamine E (mg) | 11.6 ± 4.6 | 9.9 ± 2.9 | 10.4 ± 3.6 | ns | 10.6 ± 3.8 | 12.5 ± 6.3 | ns | 8 - 13 mg |

SAFA, saturated fatty acids; MUFA, monounsaturated fatty acids; PUFA, polyunsaturated; BMS, benign course in MS; SPMS, secondary progressive MS; PPMS, primary progressive MS; kcal: kilocalories; n: number; ns: not significant; nd: not determined; SD: standard deviation

*) adjusted for Type I error (Bonferroni); **) of total energy need; a) SPMS vs BMS and vs PPMS; b) SPMS vs PPMS
